# Field-based investigation of failure modes and thresholds of granite residual soil slopes under heavy rainfall conditions

**DOI:** 10.1371/journal.pone.0317836

**Published:** 2025-02-24

**Authors:** Li Ronghua, Wu Fu, Jiang Siyi, He Minning, Pan Hongjian

**Affiliations:** Guangxi Zhuang Autonomous Region Geological Environment Monitoring Station, Nanning, China; Yogi Vemana University, INDIA

## Abstract

Rainfall is a primary coefficient of slope instability. To study patterns of slope failure mode and key index threshold values of slope instability under varying rainfall intensities, real-time data from in-situ monitoring tests of an engineering slope were utilized. This data facilitated the analysis of the temporal and spatial responses of a rainfall infiltration slope. Four field tests were conducted under four different rainfall intensities, namely 75mm/h, 125mm/h, 150mm/h, and 175mm/h, to study the progressive failure characteristics of the granite residual soil slope. These characteristics were studied based on water-time and displacement-time curves, and the rainfall duration and threshold for slope instability were proposed. The result revealed that the granite residual soil slope undergoes progressive failure under rainfall conditions, which can be categorized into four modes: “shallow local sliding”,“shallow global sliding and collapse”, “deep local sliding”, and“deep global sliding”. It takes 25 ~ 135min for the shallow sliding failure characteristics from significant deformation to instability, while the deep sliding only lasts for 18 ~ 20min. A significant correlation was observed between soil moisture content and slope instability. Instability symptoms such as cracking and peristaltic deformation begin to appear when the soil moisture content in the shallow layer of the slope increases to 42 ~ 45%. When the soil moisture content escalates to 47 ~ 50%, the slope begins to disintegrate, leading to rapid landslides and collapses.

## 1 Introduction

The mountainous and hilly areas in Guangxi Zhuang Autonomous Region of China, have a large distribution of granite and metamorphic rocks, strong weathering, and thick weathering residual layers in South China. The geological environment conditions are fragile, and it is lead to slope geological hazards [1,2]. Under the influence of strong rainfall erosion and infiltration, slopes are more prone to sudden landslides, collapse, and slope debris flows, which seriously threaten the safety of people’s lives and property [3,4]. On June 2, 2010, a landslide triggered by heavy rainfall in Rongxian County, Guangxi Province, killed 32 people, damaged 1,049 homes and caused a direct economic loss of 17.17 million [5,6]. Le T and Kubota T establishment of complex mathematical models to simulate the deformation and failure process of slopes under different rainfall intensities [7,8]. It has been found that rainfall intensity has a significant impact on slope stability, and factors such as slope gradient and soil type also affect slope stability [9,10]. Researchers have derived some quantitative relationships related to slope stability through mathematical formulas and theoretical analysis [1,11,12]. Chen J [13], Liu W [14] conducted simulated rainfall experiments on granite residual soil slope models under four different rainfall levels to study the influence of rainfall intensity and slope gradient on their failure modes. Dijkstra T A [15] and Li Hua [16] conducted outdoor field tests using artificial rainfall devices to study the stability of loess slopes under continuous rainfall conditions, measuring the moisture content, density, location of landslide shear bands, and development of cracks. This article examines the changes in slope stability under different rainfall conditions through rainfall simulation experiments on rock and soil slopes in the laboratory [17–18]. Zhan T L [19] studied the stability of unsaturated soil under rainfall conditions. Hamdhan I N [20] used FLAC3D software to explore the stability of soft rock slopes under extreme long-term rainfall conditions,and obtained the distribution characteristics and variation rules of the seepage field of the slope during the rainfall process. Hammah R E [21] used the strength reduction finite element method to analyze the sliding mode of the rock slope of a certain two-level roadside slope in the granite weathering layer of under general working conditions, groundwater seepage conditions, and rainfall infiltration conditions. Orense R P [22], Leung A K [23], and Zhang Liang [24] conducted model tests on rain-induced soil slope instability to explore the influence of rainfall characteristics on slope instability and obtain appropriate rainfall warning parameters. Gogichaishvili G P [25], Zhang Zhuo [26], and Qi Guo qing [27] considered the concept of permeability and studied the stability of rock slopes under rainfall conditions.

The above research results show that the stability analysis of slopes needs to consider numerous factors, such as rainfall intensity and the geological conditions. Therefore, to better predict slope stability and provide scientific basis for practical engineering, the rainfall duration threshold, rainfall amount threshold, and water content threshold was obtained. Different levels of rainfall intensity spray tests were vitally important, which providing reference for fine-grained investigation and analysis of slope stability, division of geological hazard risk zones, and proposing measures and plans for the prevention and control of geological hazards. It also provides key data basis for improving the accuracy of meteorological geological hazard risk warning, geological hazard monitoring, and short-term warning.

## 2 Rainfall spray test

### 2.1 Test site

The four sites under investigation are all situated on hilly slopes within the demarcated planning area of a real estate development project in Guangxi Zhuang Autonomous Region of China. The northward flowing Guangdong-Baowei Secondary Road (S215 Provincial Road) is approximately 50m west of the test area, providing relatively convenient transportation.

**Fig 1:**
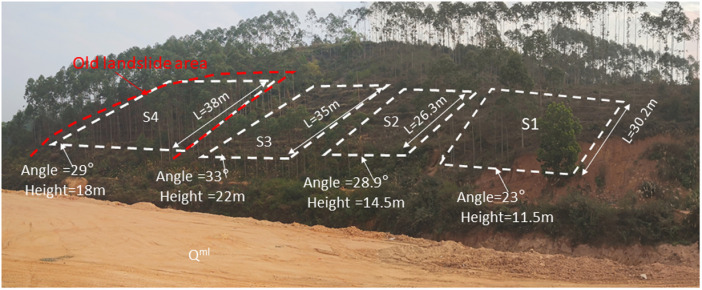
Four test sites overview.

In order to study four groups of different intensity rainfall tests, our rainfall tests were authorized by the Yulin Natural Resources Bureau. Finally, we selected four test sites, namely S1, S2, S3 and S4. Test site S1 is positioned at the southern extremity of the western slope of the longitudinally oriented hill. The lithology of the test site is silty clay, strongly weathered granite and moderately weathered granite from top to bottom. The slope height is 11.5m ~  22m.Parameters of rock and soil mass are shown in Table 1. The slope length L1 is approximately 30.2m, with an average slope of 23°, and a relative height difference of around 11.5m. Test site S2, situated 5m north of site S1, has a longitudinal slope length of 23m, a north-south width of 25m, and an area of roughly 713m². The oblique slope length, L2, is about 26.3m, the average slope is 28.9°, and the height difference is approximately 14.5m. Test site S3 has a slope length of about 35m, an average slope of 33°, and a relative elevation difference of around 22m. The base of the western edge of the site is a soil slope with a height of 3 ~ 5m. This slope was formed by past irrigation channel construction activities that involved slope cutting, and it lacks slope protection and drainage facilities. The S4 test site is primarily located on an old landslide area. The oblique slope length L4 is approximately 38m, the average slope is 29°, the relative height difference is about 18m, and the slope’s vegetation coverage rate is around 95%. Four test site overview was shown in Fig 1.

**Table 1:** Material parameters of supporting structure.

Name	Density (g/cm3)	Modulus of elasticity (MPa)	Cohesive (kPa)	Friction (°)
Silty clay	1.85	6.47	26.41	20.19
Strongly weathered granite	1.97	8.91	36.60	27.78

### 2.2 Test scheme

To study the failure modes of granite slope under different rainfall intensities, the variation characteristics of slope under four rainfall intensities, such as torrential rainstorm (175mm/h), heavy rainstorm (150mm/h), Rainstorm (125mm/h) and heavy rain (75mm/h) were analyzed. See Table 2 for rainfall intensity of each site.

**Table 2:** Rainfall intensity grade and rainfall duration in simulated test.

Serial number	Rainfall type	Rainfall intensity (mm/h) class(mm/h) (mm/h)	Duration of rainfall(min) (min)
Test site S1	Rainstorm	125	322
Test site S2	Heavy rainstorm	150	120
Test site S3	Torrential rainstorm	175	109
Test site S4	Heavy rain	75	912

**Fig 2:**
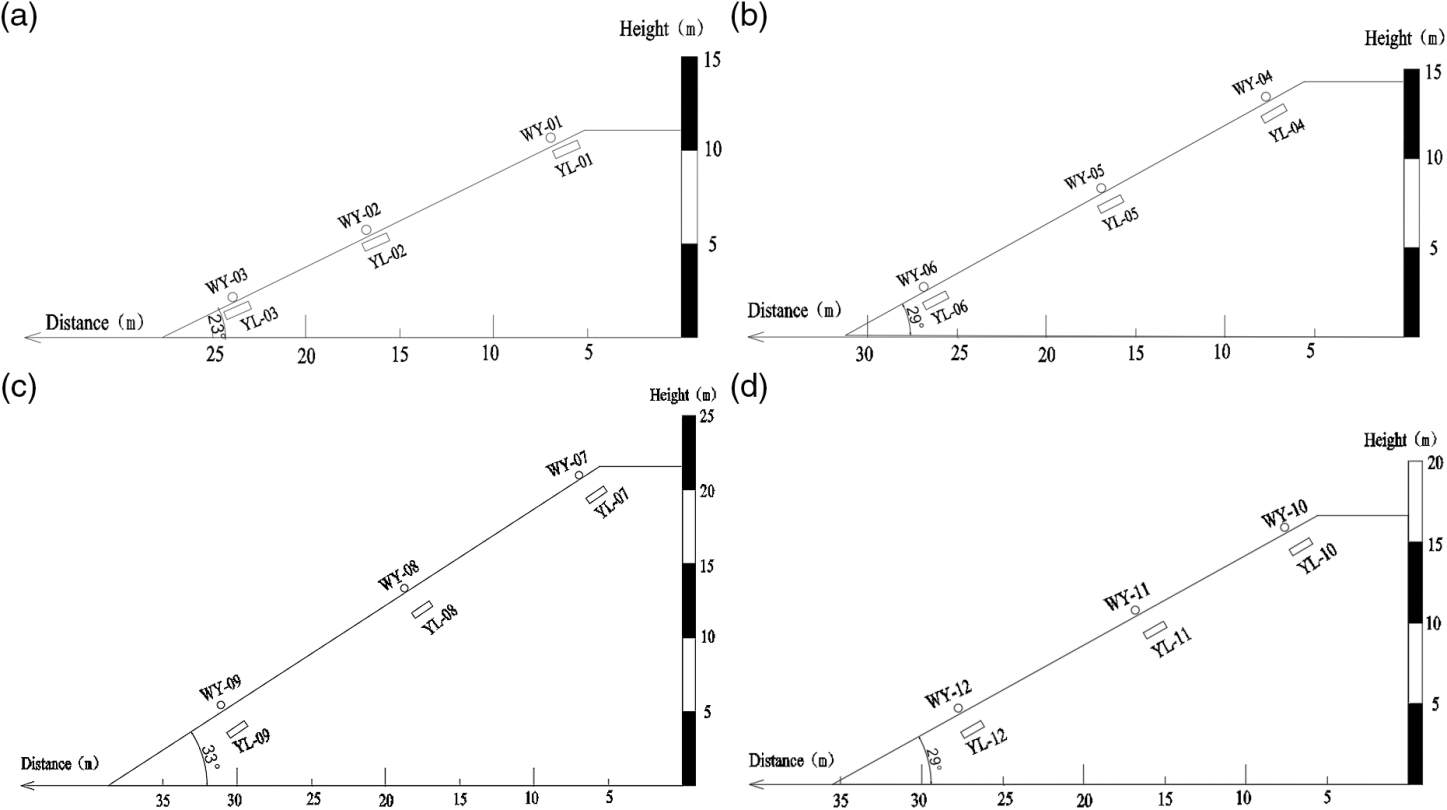
Layout of displacement measuring points and rainfall measuring points in each site (unit:m). (a) Layout diagram of measuring points in Site S1. (b) Layout diagram of measuring points in Site S2. (c) Layout diagram of measuring points in Site S3. (d) Layout diagram of measuring points in Site S4.

In order to ensure the accuracy of the test results and eliminate the boundary error, the location of each test element is selected as the middle line of each test slope. Displacement meters and soil water content monitors were installed at the top, middle, and foot of each slope, respectively. The displacement measuring points are positioned on the slope surface, while the soil water content monitors are buried at a depth of 0.8m. For Site 1, the displacement measuring points are WY-01 to WY-03, and the soil water content measuring points are YL-01 to YL-03. Similarly, for Site 2, the displacement measuring points are WY-04 to WY-06, and the water content measuring points are YL-04 to YL-06. Site 3 and Site 4 have displacement and water content measuring points ranging from WY-07 to WY-12 and YL-07 to YL-12, respectively. The specific arrangement of measuring points can be found in Table 3, while the layout of displacement measuring points for each site is illustrated in Fig 2.

**Table 3:** Layout list of measuring points in each site.

Test site name	Depth of element (m)	Water content test	Depth of element (m)	Displacement test
Test site S1	0.8	YL-01	0.0	WY-01
		YL-02		WY-02
		YL-03		WY-03
Test site S2	0.8	YL-04	0.0	WY-04
		YL-05		WY-05
		YL-06		WY-06
Test site S3	0.8	YL-07	0.0	WY-07
		YL-08		WY-08
		YL-09		WY-09
Test site S4	0.8	YL-10	0.0	WY-10
		YL-11		WY-11
		YL-12		WY-12

### 2.3 Test procedure

This test was conducted in groups, with one set of tests for each site. The test methods are roughly as follows:

1. Construction of test site. A variable-frequency submersible pump with a flow rate of 32 m^3^/h and a lift of 100 m was utilized to continuously supply water to the test site.
2. Equipment installation. A 100 kW three-phase diesel generator set with a powerful four-cylinder engine is chosen to supply electricity for the pumping equipment. The monitoring and lighting devices will be powered continuously by the 220V utility supply.
3. Equipment debugging. Liquid crystal display digital turbine flow meter and self-regulating reducing valve are used to control the water pressure and flow of each horizontal water supply pipe on the slope, so that the water pressure of each nozzle is consistent and ensure the uniform distribution of simulated rainfall.
4. Pumping spray slope. The butterfly spin sprinkler is used to rotate the water 360° to simulate rainfall, so that the droplets sprayed by the sprinkler are close to raindrops of heavy rainfall. The sprinkler head is mounted on a 2.5m high vertical pole. When the outlet water pressure is 0.15MPa, the radius of the sprinkler to evenly spray water droplets is 3m.
5. Rain gauge layout. Three rainfall monitoring stations are strategically placed at equal intervals in each test site to continuously monitor the simulated rainfall intensity. This setup allows for real-time adjustments to ensure that the simulated rainfall intensity aligns with the test requirements.
6. Displacement meter layout. Three dip acceleration monitors provided by Guilin Electronic Technology Co., LTD were used to continuously monitor slope deformation and soil moisture content change at 0.8m depth at three locations. Six GPS RTKS provided by Shanghai Huadai Navigation Technology Co., Ltd. were divided into three monitoring sections to continuously monitor the slope displacement.
7. Monitor and record slope deformation. The red-light laser rangefinder, automatic drawing steel tape measure, cable loose displacement alarm and manual regular or irregular patrol monitoring records were used to observe the local deformation of the slope.

**Fig 3:**
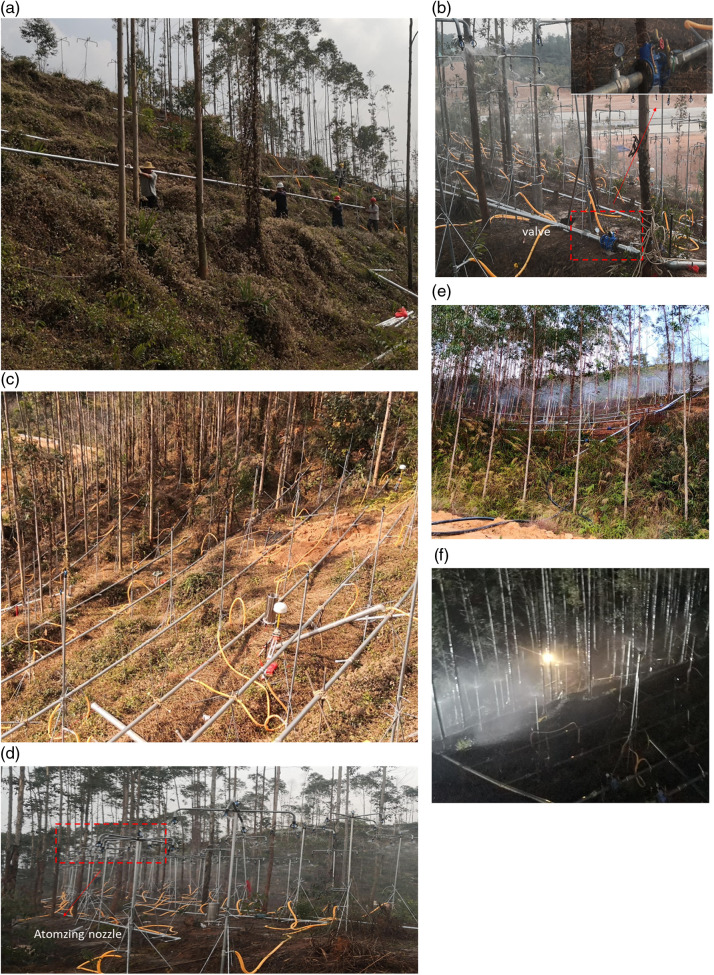
Rainfall test process. a. Construction of test site. b. Equipment installation. c. Equipment debugging. d. Rain gauge layout. e. Spray. f. Monitor and record slope.

Rainfall test process were shown in Fig 3a ~ 3f.

## 3 Analysis of test results

### 3.1 Characteristics of slope deformation instability

**Fig 4:**
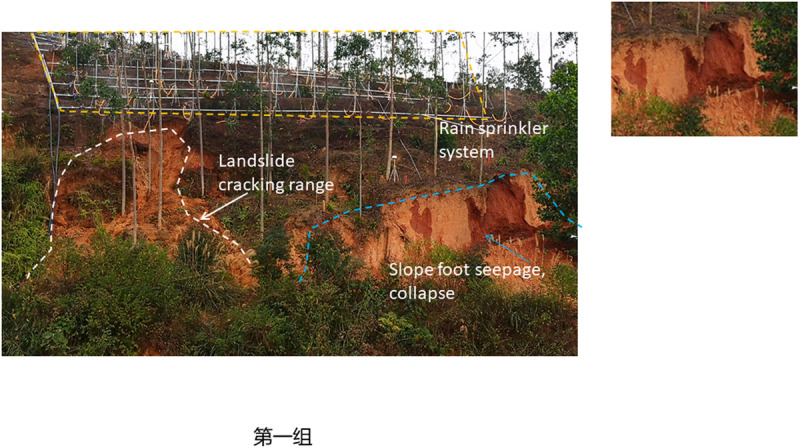
Failure characteristics of S1 slope with rainstorm (125mm/h).

**Fig 5:**
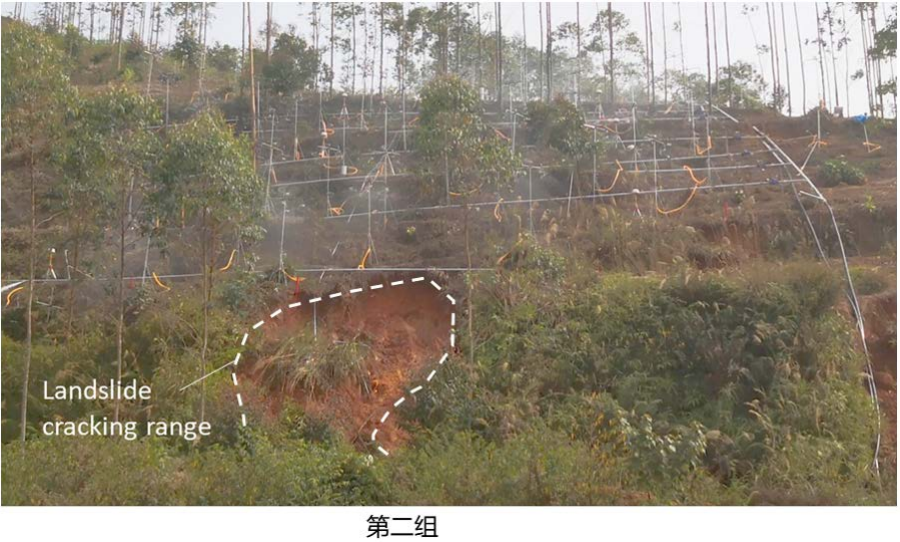
Failure characteristics of S4 slope with heavy rainstorm (150mm/h).

**Fig 6:**
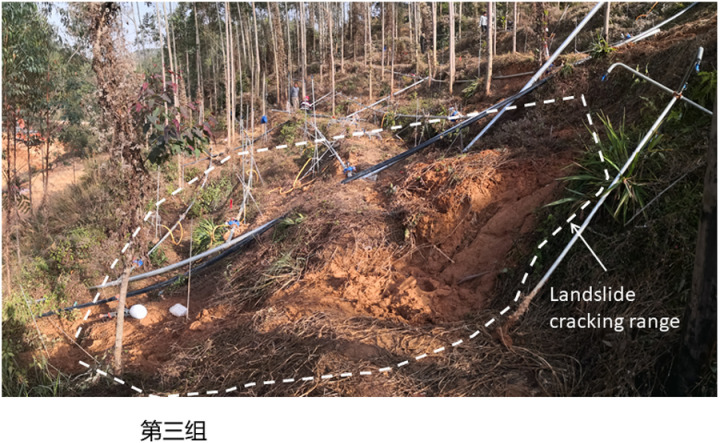
Failure characteristics of S3 slope with torrential rainstorm (175mm/h).

**Fig 7:**
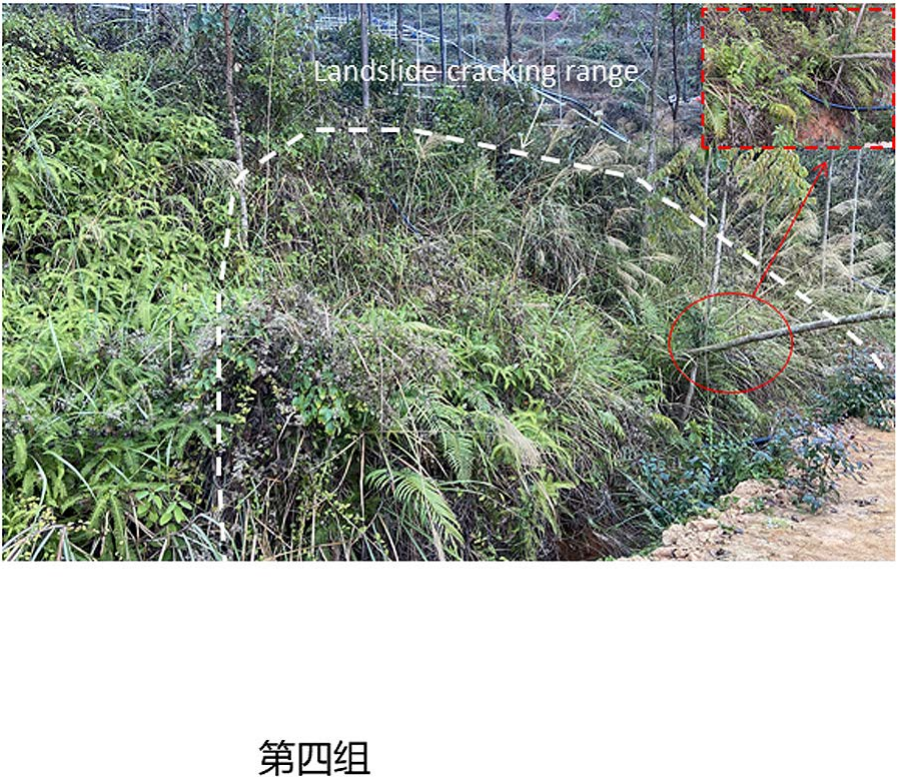
Failure characteristics of S4 slope with heavy rain (75mm/h).

To study the instability characteristics of granite residual soil slope under different rainfall intensity, the displacement and moisture content change rates of slope measurement points under different intensity conditions were monitored, and the instability characteristics of slopes under different rainfall intensity were also analyzed. The final instability characteristics of four groups of slopes (S1 ~ S4) were shown in Figs 4–7.

The four slopes with different rainfall intensities all had different failure modes, showing a progressive failure mode, and all began to deform significantly 90 minutes after the rainfall. The specific failure deformation evolution process is as follows.

As illustrated in Fig 4, during the 110-minute test period, the wet seepage area on the side slope gradually expanded. At 145 minutes, the automatic displacement monitoring instruments (WY01, WY02) sounded an alarm. Subsequently, cracks appeared and rapidly widened along the rear edge of the slope on the western side of the site. Approximately 160 minutes later, a large-scale rapid landslide occurred on the lateral slope near the crack, characterized by a “shallow overall sliding and collapse” pattern. The sliding body primarily descended along the seepage surface initially, measuring 23m in width, around 5m in length, with a thickness ranging from 0.5 to 1.5m, and estimated volume of approximately 120m^3^.

As shown in Fig 5 that after 92min of the test, both the automatic monitoring instrument and the manually set alarm started to alarm. The crack occurred at the back edge of the slope at the front of the test site, and the crack expanded rapidly. After 110min, a large-scale rapid landslide occurred on the lateral slope of the crack, and the landslide layer was the slope residual layer. The slide body is 13m wide, 8.5m long, 2.0m thick and 200m^3^ volume. It is shown as “deep local sliding mode”.

As shown in Fig 6 that at 85min of the test, the central and lower slopes of the test site began to collapse and crack, and about 2 ~  5min later, two landslides occurred and developed into slope debris flow. The landslide body is 6 ~  9m wide, 8 ~  12m long, 0.5 ~  1.0m thick and 50 ~  75m^3^ in volume. Under the erosion of the water, the two landslide bodies join to form the slope debris flow. The instability mode is “deep global slip”.

As shown in Fig 7 that around 180min of the test, the side slope began to wet and slowly permeate. At 622min, the channel slope began to slide, and the landslide layer was the slope residual layer. The slope body is 15m wide, 8.0m long, 2.0m thick and total 240m^3^ volume, showing a “shallow local sliding mode”.

In summary, different failure modes occur on slopes with four different rainfall intensities. According to the different failure modes of rainfall intensities, they were divided into four modes. They were “shallow local slip”, “shallow global slip”, “deep local slip” and “deep global slip”.

### 3.2 Law of displacement

**Fig 8:**
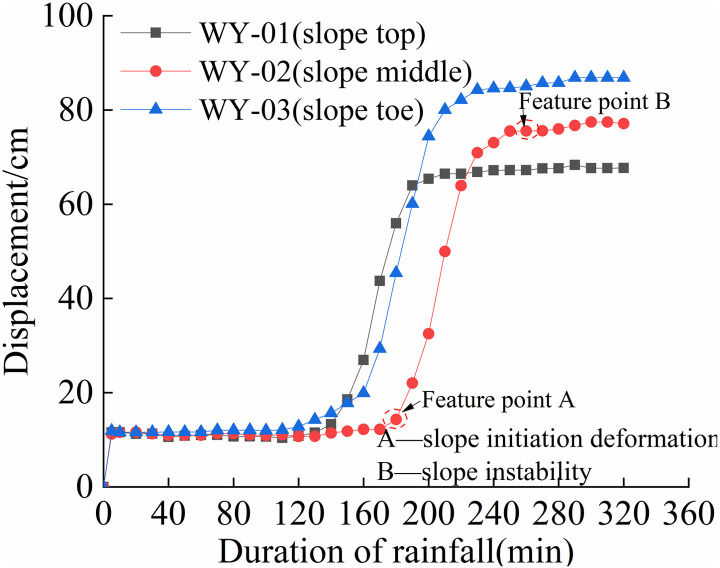
S1 Slope cumulative displacement -duration of rainfall curve (125mm/h).

The cumulative displacement at various positions of each slope varies with time, as shown in Fig 8 to Fig 11.

**Fig 9:**
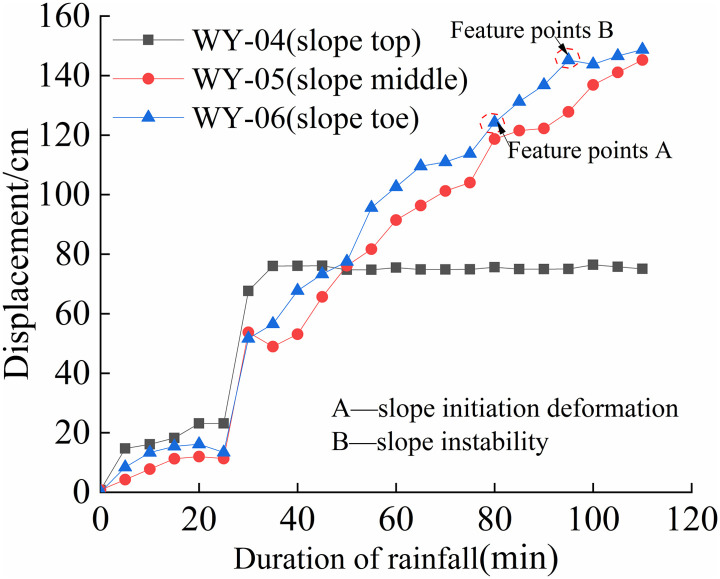
S2 Slope cumulative displacement - duration of rainfall curve (150mm/h).

**Fig 10:**
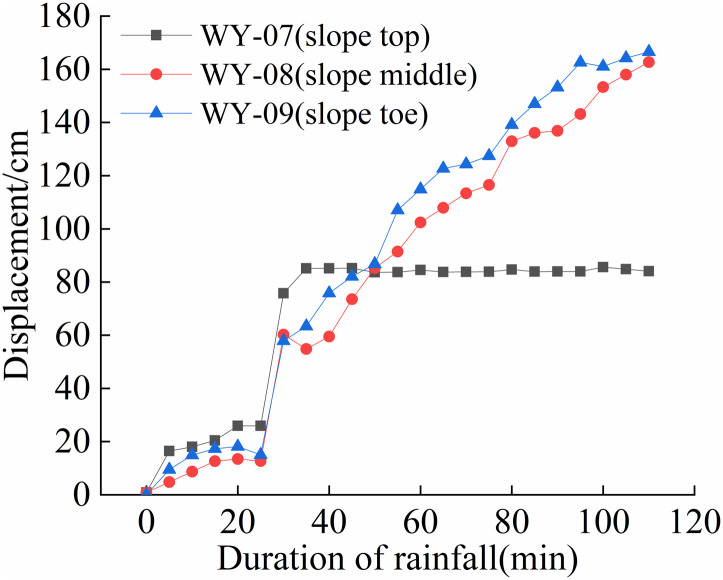
S3 Slope cumulative displacement - duration of rainfall curve (175mm/h).

**Fig 11:**
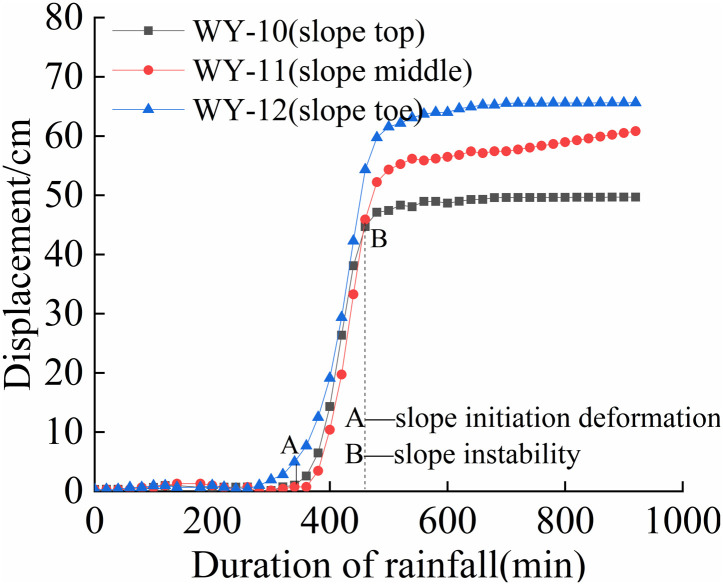
S4 Slope cumulative displacement - duration of rainfall curve (75mm/h).

As shown in Figs 8 and 11, the deformation of the four different rainfall intensities on the slope is mainly concentrated in the lower part. When the rainfall intensity is less than 125 mm/h, the displacement-time curve exhibits an S-shape. When the rainfall intensity is greater than 125 mm/h, the foot and mid-slope displacements continue to increase. From Figs 8 and 11, the cumulative displacement of the four slopes has a nonlinear relationship with time. The foot of the slope has the largest displacement while the top and mid-slope are close to each other. This is due to partial instability occurred at the foot of the slope, resulting in a sudden increase in overall displacement, followed by a slower growth rate.

From Figs 9 and 10, it can be observed that the displacement of the top of the slope increases first, followed by the mid-slope and foot of the slope almost simultaneously. As the rainfall intensity increases, the slope instability becomes more apparent, and the final displacement changes show that the foot of the slope has the largest displacement, followed by the mid-slope, and the top of the slope has the smallest displacement. In summary, the deformation of the granite residual soil slope mainly occurs at the foot of the slope. When the rainfall intensity is high, the displacement of the foot and mid-slope increases rapidly, resulting in overall sliding.

### 3.3 Law of volume moisture content

**Fig 12:**
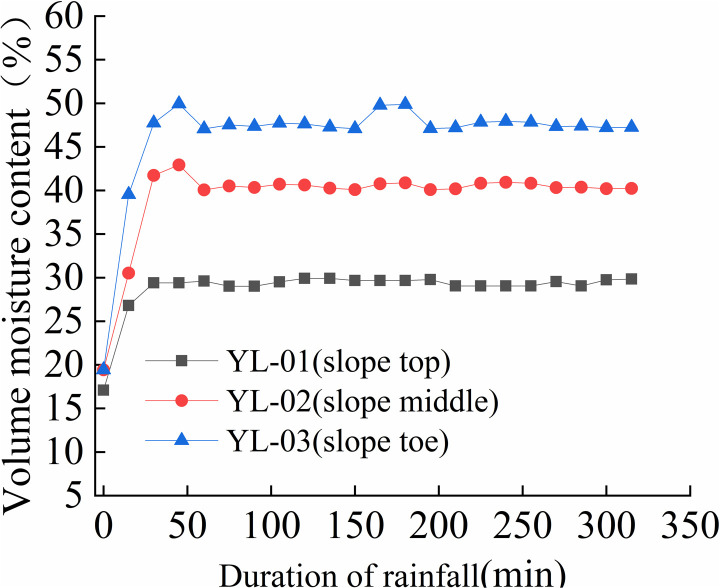
Volume moisture content-time curve of S1 slope at different positions (125mm/h).

The variation rules of volume moisture content at different positions of the slope are shown in Fig 12 to Fig 15.

As shown in Fig 12, before the spray test, the volume moisture content of YL-10, YL-11 and YL-12 remained around 20%. After 20 minutes of the test, the volume moisture content of YL-10 rapidly increased to around 30% and remained stable until the end of the test. The water content of YL-11 rapidly increased to 40% and remained stable for 55 minutes before gradually decreasing to around 27% within 60 minutes. The volume moisture content of the lower monitoring section quickly rose to around 47% in the first 27 minutes of the test and remained stable between 47% and 49% until the slope began to deform, at which point it reached 49%.

**Fig 13:**
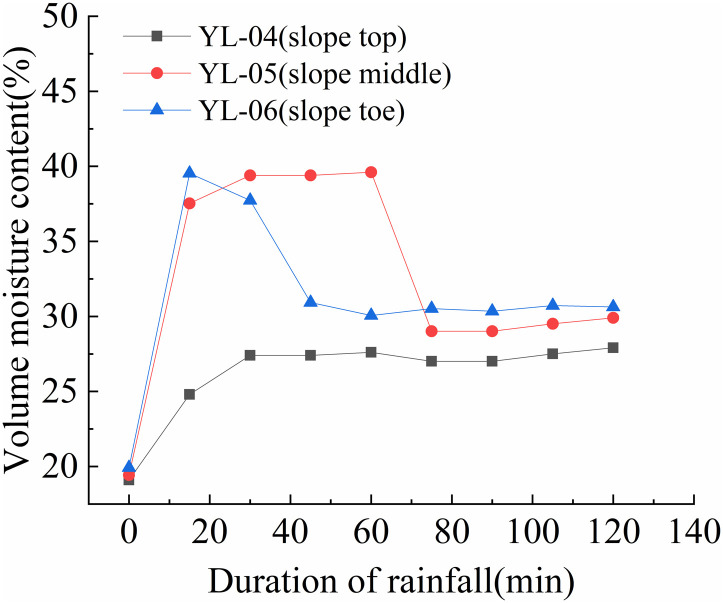
Volume moisture content-time curve of S2 slope at different positions (150mm/h).

As shown in Fig 13, before the test, the volume moisture content of all three monitoring sections remained around 20%. The volume moisture content of the upper monitoring section quickly rose to around 30% in the first 20 minutes of the test and remained stable until the end. The volume moisture content of the middle monitoring section rapidly increased to around 40% in the first 15 minutes and remained stable for 55 minutes before gradually decreasing to around 27% within 60 minutes and remaining stable until the spray test end. The volume moisture content of the lower monitoring section quickly rose to around 47% in the first 27 minutes of the experiment and remained stable between 47% and 49% until the slope began to deformation, at which point it reached 49%. After 180 minutes, the volume moisture content began to rapidly decrease to around 30% and remained stable until the test end.

**Fig 14:**
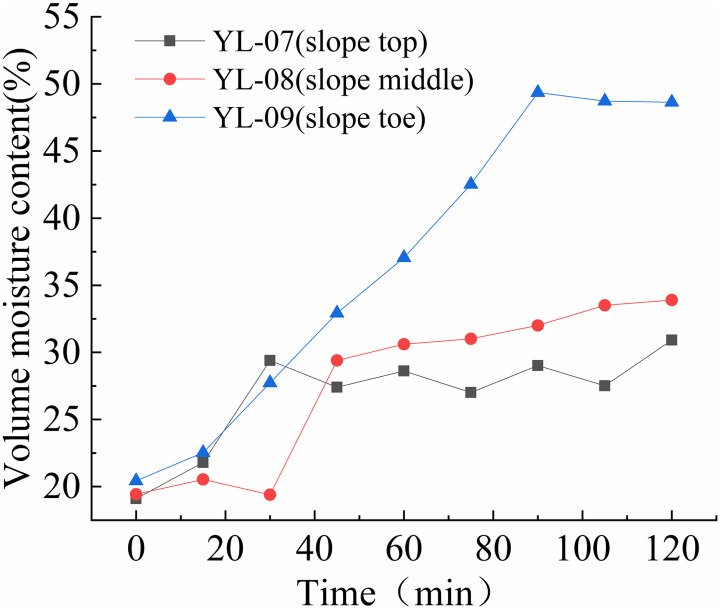
Volume moisture content-time curve of S3 slope at different positions (175mm/h).

As shown in Fig 14, before the spray test, volume moisture content of both monitoring sections remained between 17% and 22%. The soil moisture content of the upper monitoring section remained around 20% in the first 15 minutes of the test, then quickly rose to around 29% within 12 minutes, and remained between 26% and 30% with minor fluctuations for the next 26 hours. The volume moisture content of the middle monitoring section remained around 20% for the first 39 minutes of the experiment, then quickly rose to around 30% within 23 minutes, and remained between 30% and 34% with minor fluctuations for the next 20 hours. The water content of the lower monitoring section remained around 22% for the first 15 minutes of the experiment, then gradually increased to between 47% and 49% within one hour.

**Fig 15:**
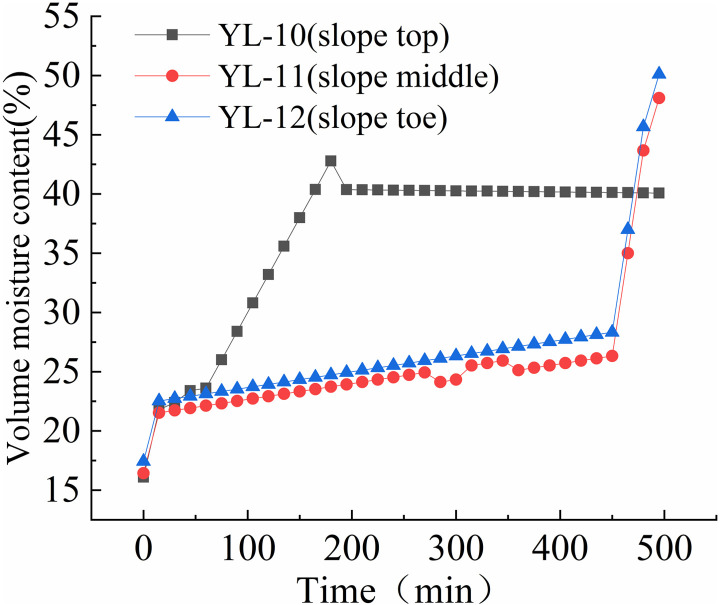
Volume moisture content-time curve of S4 slope at different positions (75mm/h).

As shown in Fig 15, before the spray test, volume moisture content of all three monitoring sections remained between 18% and 23%. The volume moisture content of the upper monitoring section gradually increased to around 40% within the first two hours of the test and remained stable until the end. The volume moisture content of the middle and lower monitoring sections gradually increased from 17% to around 28% within the first 450 minutes of the test, then quickly rose to around 50% within 45 minutes. In summary, the volume moisture content of the slope was highest at the foot of the slope, followed by the middle section, and lowest at the top of the slope. Under rainfall, the slope’s soil moisture content rapidly increased within the first 20 minutes, with the upper and middle sections increasing at a significantly slower rate than the lower section. The water content of the shallow soil on the slope’s lower section increased rapidly, reaching 40% within 30 minutes, and reaching between 42% and 50% within one to two hours under rainfall intensities greater than 125mm/h. When the volume moisture content of the shallow soil on the slope reached between 42% and 45%, signs of instability such as cracking and creeping deformation appeared on the slope. When the volume moisture content reached between 47% and 50%, the slope began to collapse and rapidly lead to landslides and collapses. Under the scouring action of surface water flow, these events can develop into debris flows on the slope.

### 3.4 Key index of rainfall threshold

**Fig 16:**
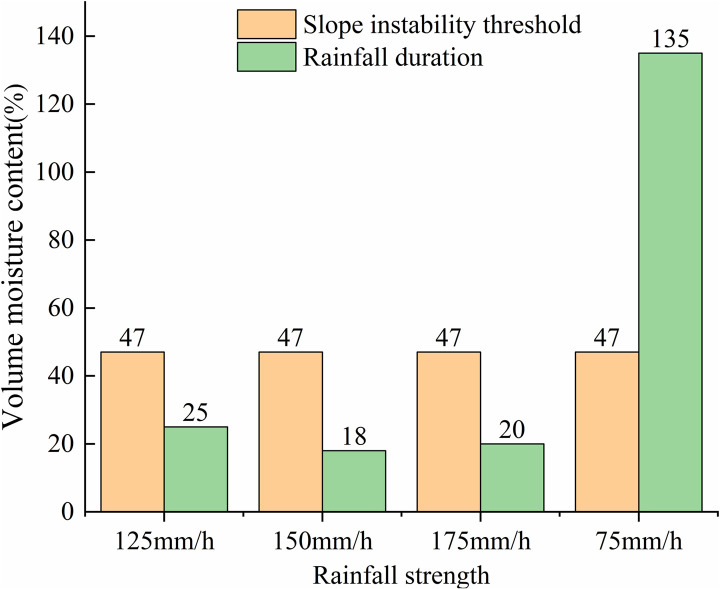
Key indexes of granite residual soil slope under different rainfall intensities.

According to the above four groups of test results, the threshold values of key indicators such as rainfall time, rainfall and soil moisture content corresponding to slope instability caused by continuous rainfall of four rainfall intensity levels obtained in this test are shown in Table 4 respectively. The change rule of key indexes of granite residual soil slope under different rainfall intensities is shown in Fig 16.

**Table 4:** Key index threshold values of slope instability caused by continuous rainfall intensity.

Rainfall strength (mm/h)	Threshold of slope initiation deformation			Slope instability threshold			Duration Time (min)
	Duration (min)	Rainfall (mm)	Water content (%)	Duration (min)	Rainfall (mm)	Water content (%)	
125	145	298	42	160	329	47	15
150	92	230	42	110	275	47	18
175	85	219	42	105	306	47	20
75	367	609	42	502	778	47	135

As illustrated in Fig 16, there is no significant correlation between the threshold moisture content at the onset of slope deformation and the threshold moisture content at complete instability, in relation to rainfall intensity. However, the duration of slope deformation is significantly influenced by rainfall intensity. Under varying rainfall intensities ranging from 75mm/h to 175mm/h, the limiting water content for slope deformation and instability deformation are 42% and 47%, respectively. Notably, when the rainfall intensity exceeds 125mm/h, the period of landslide instability and failure is merely 20 minutes. Conversely, at rainfall intensity of 75mm/h, the duration of slope instability and deformation extends to 140 minutes.

## 4 Discussion

The four sites rainfall spray test achieved a relatively ideal effect. However, due to the complexity and systematizes of the site test, there are still some deficiencies in this test, mainly as follows:

1. Affected by the insufficient automatic monitoring equipment, the density of displacement monitoring, soil moisture content monitoring and incline monitoring equipment deployed is too small to comprehensively monitor the whole process of slope deformation. It was found that, in the test process, all test sites began to deform in a small local range. Due to the small initial deformation range, there was no automatic monitoring equipment to detect it, and the slope instability deformation time threshold obtained by the test lagged 3 ~  20 minutes.
2. Limited by equipment accuracy, water content monitoring at different depths at the same point could not be carried out, rainfall infiltration depths of granite and granite-metamorphic rock areas under different rainfall intensities could not be understood, and soil thickness of slope surface landslide and debris flow on the slope under different rainfall intensities could not be determined.
3. It was difficult to obtain soil moisture content and slope deformation data at each time, due to the lack of multifunctional monitoring instrument integrating soil moisture content, incline acceleration and three-coordinate positioning instrument.
4. As the test time was in the dry season, the moisture content of soil is low, which may cause the rainfall duration of geological disasters to be longer.

## 5 Conclusion

In order to study the stability of granite metamorphic rock slope, classify the risk areas of geological disasters, and put forward the prevention and control measures and schemes of geological disasters, the instability tests of granite metamorphic rock slope under different rainfall intensity conditions were carried out to analyze the variation rules of soil moisture content and slope displacement under rainfall duration, and the following conclusions were obtained.

1. There is a significant correlation between slope instability and soil moisture content. When the volume moisture content of the slope rises to 42 ~  45%, the slope begins to show signs of instability such as cracking and peristaltic deformation. When the volume moisture content rises to 47 ~  50%, the slope begins to disintegrate and landslides and collapses quickly.
2. Rainfall intensity is significantly correlated with slope failure model. When rainfall intensity is 75mm/h, the slope instability mode presents shallow sliding mode. However, rainfall intensity is 150mm/h, the slope instability mode presents deep sliding mode.
3. The correlation degree between the threshold moisture content of slope deformation at the beginning and the threshold moisture content of deformation of complete instability and rainfall intensity is not significant, while the duration of slope deformation is significantly affected by rainfall intensity. When the rainfall intensity exceeds 125mm/h, the time of landslide instability and failure only lasts for 20min, and when the rainfall intensity is 75mm/h, the time of slope instability and deformation is 140min.
4. When the rainfall intensity is 75mm/h, the rainfall reaches 609mm, when the rainfall intensity is 125mm/h, the rainfall reaches 298mm, the slope begins to lose stability, when the rainfall intensity is 175mm/h, the rainfall reaches 219mm, the slope deformation is suggested in the rainy season, the rainfall intensity is 219mm.
